# Management of Groin Pain Using an Iliohypogastric Nerve Block in a Patient with Inguinal Hernia due to Persistent Müllerian Duct Syndrome

**DOI:** 10.1155/2021/7577632

**Published:** 2021-08-12

**Authors:** Takanori Sekito, Takuya Sadahira, Masahiro Sugihara, Kohei Edamura, Motoo Araki, Yasutomo Nasu

**Affiliations:** ^1^Department of Urology, Dentistry and Pharmaceutical Sciences, Okayama University Graduate School of Medicine, 2-5-1 Shikata-cho, Kita-ku, Okayama 700-8558, Japan; ^2^Division of Surgery, Nishi Fukuyama Hospital, 340-1 Matsunagacho, Fukuyama City, Hiroshima 729-0104, Japan

## Abstract

Persistent Müllerian duct syndrome can cause an inguinal hernia, although this is a rare occurrence; recurrent inguinal hernias can, in turn, cause ongoing groin pain. Management of groin pain plays an important role in patients' quality of life. We present our experience with a 43-year-old man who had a 2-week history of left-sided groin pain. The patient underwent laparoscopic surgery for a left inguinal hernia via the transabdominal preperitoneal approach. Right-sided cryptorchidism was noted during surgery, with a solid structure—thought to be a uterus—extending into the left inguinal canal. The diagnosis was persistent Müllerian duct syndrome, and the groin pain was relieved after a laparoscopic right orchiectomy with a bilateral preperitoneal hernia repair using a mesh. Four years later, magnetic resonance imaging performed for new-onset left groin pain showed a left inguinal hernia caused by the uterine structure. We diagnosed the recurrent hernia as the cause of his pain. Prior to performing any invasive surgical procedures, an iliohypogastric nerve block was performed using 1% lidocaine. Short-term analgesia was provided by the block, improving his quality of life. He has been followed since then and has declined surgical neurectomy. An iliohypogastric nerve block can be an effective method of controlling groin pain caused by an inguinal hernia resulting from persistent Müllerian duct syndrome; the effectiveness of the nerve block will help determine whether surgical neurectomy is indicated for permanent pain control.

## 1. Introduction

Persistent Müllerian duct syndrome (PMDS) is characterized by the presence of Müllerian remnants in patients with a 46,XY karyotype and normal male external genitalia [1]. The diagnosis of PMDS is usually made when the Müllerian structures are detected incidentally during investigation or treatment for inguinal hernia, with or without cryptorchidism [2]. Surgical management and follow-up for patients with PMDS remain controversial and challenging because of 2 major complications: infertility and malignant degeneration of the testis.

There are rare reports of groin pain induced by an inguinal hernia resulting from PMDS [3]. Pain management is an important aspect of care for patients with PMDS. We report our experience with a patient who had a recurrent inguinal hernia caused by PMDS, resulting in persistent groin pain. We successfully managed this patient's pain with an iliohypogastric nerve block. This effective management technique will help clinicians to decide whether surgical neurectomy will be indicated as the next step for pain control.

## 2. Case Presentation

A 43-year-old man presented to our institution with a 2-week history of left-sided groin pain. He was married but had no children and had initiated treatment for infertility at another institution three years prior. The semen analysis revealed oligozoospermia. His brother had a history of cryptorchidism and eventually developed testicular cancer and underwent orchiectomy. On physical examination, the right testis was not palpable in the scrotum, and there was a nonreducible swelling at the left groin. Computed tomography demonstrated herniation in the left groin and right cryptorchidism. The patient desired removal of the right-sided cryptorchidism because of his family history of testicular cancer.

**Figure 1:**
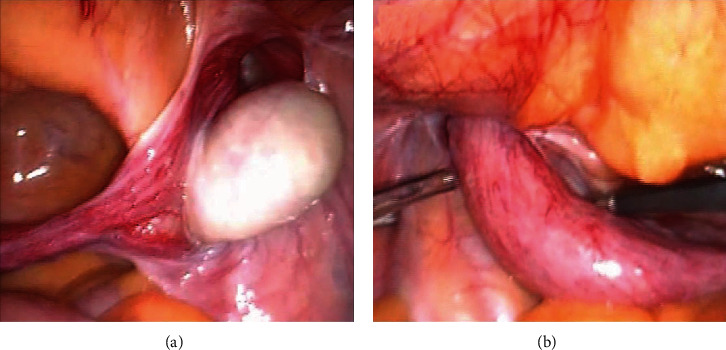
(a) Right-sided cryptorchidism is seen during laparoscopic surgery for a left inguinal hernia. (b) Laparoscopic inspection reveals a solid structure, resembling a uterus, extending into the left inguinal canal.

We performed herniorrhaphy using the transabdominal preperitoneal approach for the left inguinal hernia. We confirmed right cryptorchidism (Figure 1(a)) at the time of surgery. We also noted a solid structure resembling a uterus that extended into the left inguinal canal (Figure 1(b)). We performed a laparoscopic right orchiectomy with bilateral preperitoneal hernia repair using a mesh. The structure that resembled a uterus was not removed to preserve the remaining internal male genitalia. Histopathology of the right testis showed no evidence of malignant degeneration. The patient was discharged without groin pain or any other complications after surgery and was advised of his diagnosis of PMDS.

**Figure 2:**
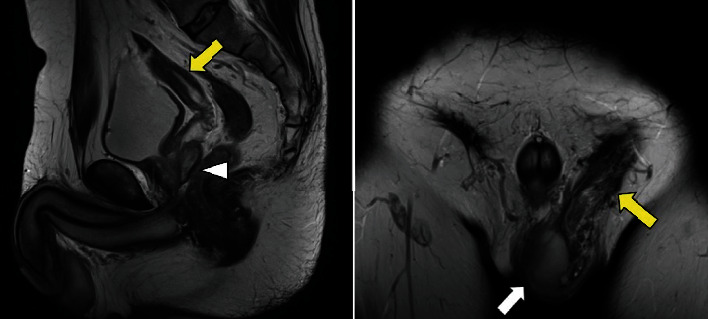
Magnetic resonance imaging shows a left inguinal hernia containing uterine and vaginal structures extending from the left inguinal canal into the scrotum (yellow arrows). There is a normal testis in the left hemiscrotum (white arrow). Müllerian duct derivatives are connected to the prostate on the dorsal side (white arrowhead).

Four years later, he returned with new-onset left groin pain that had persisted for 3 weeks, accompanied by left lower quadrant abdominal pain. The pain was causing difficulty with his occupation as a truck driver. Magnetic resonance imaging (MRI) showed a left inguinal hernia, with uterine and vaginal structures extending from the left inguinal canal into the scrotum. The Müllerian duct derivatives were continuously connected to the prostate on the dorsal side (Figure 2). At the time of his arrival, the patient eventually did not conceive a child even after in vitro fertilization for several times as fertility treatment; therefore, the patient already decided to discontinue the fertility treatments.

We diagnosed the recurrent hernia as the cause of his pain. Prior to performing any invasive surgical procedures, we performed an iliohypogastric nerve block using 1% lidocaine. This nerve block relieved his pain. He has had an uneventful course since the block and has been able to return to work. Two weeks after the nerve block, the patient returned to our hospital for follow-up care and had no complaint of groin pain. We discussed how to manage recurrent groin pain in the future. Our plan for any future recurrence was to perform surgical neurectomy of the iliohypogastric nerve for long-term pain control and better quality of life. However, the patient did not agree to undergo the procedure at this most recent appointment.

## 3. Discussion

The rare condition of PMDS occurs when there is either a deficiency of antimüllerian hormone (AMH) activity or an abnormality in AMH receptors. Sertoli cells in the fetal testicle produce AMH, causing the involution of embryonic Müllerian structures in normal male fetuses [4]. The 3 main clinical presentations of PMDS are bilateral cryptorchidism, hernia uteri inguinalis, and transverse testicular ectopia. One review notes that 59% of patients present with bilateral cryptorchidism, 19% with hernia uteri inguinalis, and 22% with transverse testicular ectopia [5].

Hernia uteri inguinalis occurs when a single testis remains in the abdomen while the other is in the inguinal canal, along with the uterus and ipsilateral fallopian tube, resulting in unilateral cryptorchidism with a contralateral inguinal hernia [4]. This type occurs in 14% of patients with idiopathic PMDS and approximately 20% of patients with mutations involving the AMH pathway [5]. Although our patient had this presentation, we did not perform karyotype analysis to detect mutations in the AMH pathway.

Malignant degeneration and infertility are major potential complications of PMDS. We performed a laparoscopic orchiectomy of the undescended testicle, considering our patient's family history of testicular cancer. However, because he was also trying to father a child at the time of the surgery, it was important to leave the remaining, correctly positioned, testis in place [6].

How to manage fertility in a patient with PMDS is important. In one review, intracytoplasmic sperm injection may be helpful in some cases with PMDS as fertility treatment [5], like what was performed to our patient. Infertility in PMDS can be related to the structural abnormalities caused by the Müllerian organs or long-term cryptorchidism [1]. However, an attempt to remove the Müllerian structures can damage the male excretory ducts enclosed in the Müllerian derivatives [5]. Minimizing risk to damage vital structures for fertility should be considered before a complete excision of the Müllerian organs. To the best of our knowledge, although there are no reports describing about freezing sperm in patients with PMDS before surgery, a patient can also be advised to freeze sperm before the Müllerian organs will be removed.

In PMDS, the histology of the testes is usually normal. The incidence of malignant degeneration in these testes is 18%, whose rate is the same as the rate of malignancy in abdominal testes in men without PMDS [4]. Concerning resection outcomes of Müllerian organs, Müllerian malignancies are much less frequent than testicular malignancies [1]. Literature on this topic is rare, with a few reports describing uneventful recovery after surgery in a small number of patients [2–4].

**Figure 3:**
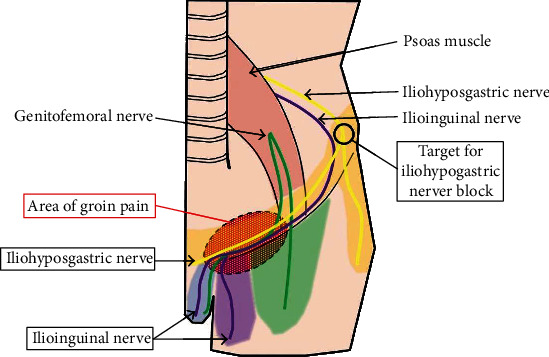
Sensory nervous distribution in the groin area. The dashed ellipse represents the area of our patient's groin pain. The circle represents the area of iliohypogastric nerve block placement.

Groin pain induced by an inguinal hernia, itself caused by PMDS, has been rarely reported [7]. The inguinal region receives sensory innervation from the iliohypogastric, ilioinguinal, and genitofemoral nerves [8], as shown in Figure 3. The iliohypogastric nerve predominantly originates from L1 [9] and supplies the skin of the pubic area and the upper part of the thigh and buttock. Our patient experienced groin pain when his internal uterine and vaginal structures extended into the left inguinal canal. When the structures were released from the inguinal canal for some reason, e.g., body movement, he experienced spontaneous relief. However, when the structures extending into the left inguinal canal became incarcerated, they caused persistent groin pain accompanied by left lower quadrant abdominal pain; this pain resulted from compression of the iliohypogastric nerve.

It is also possible that entrapment neuropathy caused our patient's pain. The pain caused by entrapment neuropathy starts within weeks, and neurological symptoms may occur. Repeated nerve blocks are usually needed until the pain resolves [10]. Our patient's pain started 4 years after his orchiectomy and hernia repair, and he had no neurological symptoms. Additionally, given the MRI findings, we suspected that his pain was caused by a hernia resulting from PMDS.

Previous studies show that the branches of the iliohypogastric, ilioinguinal, and genitofemoral nerves communicate freely and that there is great variation in the sensory innervation of the inguinal region [8]. One report demonstrates that the iliohypogastric nerve communicates with accessory branches of the ilioinguinal nerve (55%) [9]. The ilioinguinal nerve runs parallel to the spermatic cord; therefore, an iliohypogastric nerve block may have some effect in improving groin pain in the sensory innervation region of the ilioinguinal nerve. Performing a simultaneous ilioinguinal and iliohypogastric nerve block could be an even better option to control groin pain.

The use of an iliohypogastric nerve block provided good pain control for our patient, but this relief will most likely be temporary. Definitive surgical treatment would involve resection of the Müllerian organs, i.e., hysterectomy and salpingectomy. Resection of Müllerian organs is an invasive procedure and carries the risk of damaging the vas deferens and disrupting the blood supply to the testes [11, 12]. Our plan was to offer surgical neurectomy of the iliohypogastric nerve as a less invasive alternative to control recurrent groin pain, since we confirmed that the iliohypogastric nerve block was effective. However, our patient did not agree to this plan. To appropriately manage pain resulting from PMDS, it is extremely important to take a patient's full clinical picture into consideration. Nerve blocks can be helpful in providing immediate pain control, and they provide information when considering subsequent optimal treatment.

## 4. Conclusion

Management of groin pain is an important element of quality of life for patients with inguinal hernias due to PMDS. Use of an iliohypogastric nerve block can be a helpful option to control this pain, and the effectiveness of the nerve block will help clinicians to decide whether surgical neurectomy will be indicated as subsequent treatment for pain control.

## Data Availability

The data used to support the findings of this study are included within the article.
